# Diagnostic value of end tidal capnography in patients with hyperglycemia in the emergency department

**DOI:** 10.1186/s12873-016-0072-7

**Published:** 2016-01-29

**Authors:** Ralphe Bou Chebl, Bryan Madden, Justin Belsky, Elie Harmouche, Lenar Yessayan

**Affiliations:** Department of Emergency Medicine, American University of Beirut, Beirut, Lebanon; Department of Emergency Medicine, Henry Ford Hospital, Detroit, MI USA; Department of Emergency Medicine, Massachusetts General Hospital, Boston, MA USA; Division of Nephrology and Hypertension, Henry Ford Hospital, Detroit, MI USA; Division of Pulmonary and Critical Care Medicine, Henry Ford Hospital, Detroit, MI USA

**Keywords:** Capnography, Diabetic ketoacidosis, Emergency department

## Abstract

**Background:**

Diabetic Ketoacidosis (DKA) is a potentially life-threatening emergency that requires prompt diagnosis and treatment. In paediatric populations an end tidal capnography value greater than 36 mmHg was found to be 100 % sensitive in ruling out DKA.

**Methods:**

A cross sectional observational study of adults ≥ 17 years of age presenting to the emergency department between January 2014 and May 2014 with glucose > 550 mg/dL. In all patients, nasal capnography and venous blood gas analysis were performed prior to any insulin or intravenous fluid administration. The diagnosis of DKA was based on the presence of anion gap metabolic acidosis, hyperglycaemia and ketonemia. The overall diagnostic performance (area under the curve [AUC]), sensitivity, specificity and likelihood ratios at different end tidal CO_2_ (ETCO_2_) cut-offs were determined.

**Results:**

71 patients were enrolled in the study of which 21 (30 %) met the diagnosis of DKA. The area under the curve for ETCO_2_ was 0.95 with a 95 % CI of 0.91 to 0.99. Test sensitivity for DKA at ETCO_2_ level ≥35 mmHg was 100 % (95 % CI, 83.9–100). An ETCO_2_ level ≤ 21 mmHg was 100 % specific (95 % CI, 92.9–100.0) for DKA.

**Conclusion:**

Nasal capnography exhibits favourable diagnostic performance in detecting patients with or without DKA among those who present to the emergency department with a glucometer reading > 550 mg/dL.

## Background

Diabetes mellitus is the most common endocrine disease in the world. Acute complications of diabetes include severe hyperglycaemia, diabetic ketoacidosis (DKA) and hyperosmolar hyperglycaemic state (HHS). DKA is a potentially life-threatening emergency characterized by hyperglycaemia, metabolic acidosis and ketonemia along with severe electrolyte abnormalities [1]. HHS is defined as a state of severe hyperglycaemia without ketosis, with a glucose >600 mg/dL but with levels frequently exceeding 1000 mg/d. HHS is also characterized by neurological impairment, with the presentation ranging from altered mental status to stupor. All hyperglycaemic states require fluid resuscitation. The administration of regular insulin to DKA patients via continuous intravenous infusion or by frequent subcutaneous or intramuscular injections is the mainstay of treatment and is needed to stop the breakdown of fatty acids into ketones and to revert the acidotic state. While insulin is not harmful in severe hyperglycemia, patients may achieve euglycaemia with fluid resuscitation alone and an insulin drip is not needed [2]. Capnography provides an indirect means to detect metabolic acidosis. It monitors the partial pressure of carbon dioxide (CO_2_) in respiratory gases. The partial pressure of CO_2_ at end expiration is termed end tidal CO_2_ (ETCO_2_) [3]. In response to the decreased serum pH that is associated with a decrease in serum bicarbonate, an increase would occur in alveolar minute ventilation with a corresponding decrease in arterial CO_2_ tension. Because ETCO_2_ closely approximates arterial CO_2_ tension, it can potentially be used to predict the presence or absence of DKA. Obtaining blood tests may be time-consuming. A rapid non-invasive sensitive screening tool that rules out DKA in patients with severe hyperglycaemia may avoid unnecessary administration of insulin infusion as part of initial therapy. Similarly, a non-invasive test with high specificity in identifying those with DKA; may expedite prompt diagnosis and treatment of DKA.

### Goals of this investigation

In our emergency department, patients found to have blood glucose >550 mg/dL, regardless of clinical status, are taken to our high acuity area. This triage process often leads to overcrowding the critical area and overwhelming the medical team. For this subset of patients, we wished to investigate the diagnostic characteristics of ETCO_2_ to assess patients for DKA versus severe hyperglycaemia. We hypothesized that ETCO_2_ exhibited at least a “fair discriminatory power” (i.e., 0.7 < AUC <0.8) [4] for DKA in patients with blood glucose >550 mg/dL. At 80 % power, alpha = 0.05, a ratio of sample sizes in negative to positive groups with DKA of 4, and an area under the receiver operating curve (ROC) of 0.75 (fair discriminatory power) that is significant from the null hypothesis value of 0.5 (no discriminatory power), a minimum of 64 patients were required for the study. To include at least this number of patients, a 6-month inclusion period was considered. The objectives of this study were to characterize the overall diagnostic performance (area under the curve [AUC]) of ETCO_2_ in patients with blood glucose > 550 mg/dL (30.6 mmol/L); to determine the sensitivity, specificity, likelihood ratios at different ETCO_2_ cut-offs; and to establish the degree of correlation of both venous pH and serum bicarbonate with ETCO_2_, and between venous pH and serum bicarbonate.

## Methods

### Study design and setting

Cross sectional observational study was conducted at a single emergency department in an urban tertiary care centre in Detroit, Michigan, between January 2014 and May 2014. Given that there was no harm or injury in applying an end tidal monitor to patients’ nasal cannula, a waiver of informed consent was granted. This study was approved by our hospital’s institutional review board (IRB #8560).

### Selection of participants

As per our emergency department’s protocol, patients with a known history of diabetes, or patients presenting with specific complaints (generalized weakness, vomiting, diaphoresis, altered mental status, polyuria) are screened at triage with a point of care glucose measurement. Inclusion criterion included a point of care glucometer (Accu-Chek Inform II, Roche, Basel, Switzerland) reading of “high” on presentation which corresponds to a glucose level >550 mg/dL. Exclusion criteria consisted of receipt of intravenous fluids or insulin prior to the application of nasal capnography.

### Methods and measurements

Patients were approached and identified by the nursing staff in triage. Patient recruitment and enrolment occurred at all times throughout the duration of the study. All Eligible patients had nasal CO_2_ sampling cannula placed by trained medical personnel while they were being placed on the monitor and having their initial vital signs taken. The cannula was attached to a capnograph/capnometer (Microstream EtCO_2_ consumable, Smart Capnoline, Philips Healthcare, Andover, MA). A single ETCO_2_ value was documented when a stable waveform was recorded on the monitor (Philips, intellivue MX700, Philips healthcare, Andover, MA). Venous blood was collected into collecting tubes for electrolytes, glucose, beta hydroxybutyrate (ßHB), and complete blood count. No time delays between the blood draws and the application of the end tidal capnography. A venous blood gas (VBG) sample was then collected in an arterial blood collection syringe, sent to the laboratory over ice and analysed by blood gas analyser (ABL800 Flex, Radiometer America Inc., Westlake, OH). Measurements of interest were pH and CO_2_ obtained from the VBG sample and measured serum bicarbonate (HCO3), ßHB, and blood glucose from routine analysis of venous blood. Capnography was compared to a reference standard for diagnosing DKA defined as a triad of hyperglycaemia >250 mg/dL, high anion gap metabolic acidosis and ketonemia [5]. Ketonemia was defined as ßHB level >3 mmol/l. The high anion gap acidosis was defined as having an anion gap >12 with either one of the following criteria; a serum bicarbonate ≤ 18 mmol/L or a blood pH < 7.30. Given that the inclusion criteria was a glucose >550 mg/dL, all patients met the hyperglycaemia criterion.

### Analysis

Statistical analysis was performed using SAS software version 9.3 (SAS Institute, Inc., Cary, NC) and ROCs were performed using MedCalc Statistical Software version 13.2.2 (MedCalc Software bvba, Ostend, Belgium). Categorical variables were presented as frequencies and percentages. Continuous variables were intentionally presented as median, interquartile range (IQR), minimum and maximum to appreciate the spread of the data. Mann Whitney rank sum test was used for group comparisons for continuous data. Categorical data comparisons were performed using chi-square test. Pearson coefficients were used to evaluate the correlation between ETCO_2_ and other arterial blood measurements and between venous pH and serum bicarbonate. Sensitivities and specificities were calculated at different levels of ETCO_2_. For all analyses, *P* < .05 was considered significant.

## Results

### Characteristics of study patients

Seventy-one consecutive patients were enrolled in the study. The median age was 48.0 years (IQR 39–58) and 39 (55 %) were male. All patients had a known history of diabetes. Twenty-one patients had DKA (prevalence 30 %). There was a significantly higher percentage of insulin dependent diabetic patients in the DKA group (57 % versus 24 %). Patient demographics, by presence or absence of DKA are presented in Table 1. Laboratory and diagnostic values are presented in Table 2. ETCO_2_ values ranged between 5 and 35 mmHg in patients with DKA. Patients with DKA had higher median levels of glucose and ßHB and lower partial pressure of CO_2_ (pCO_2_), ETCO_2_, serum bicarbonate, and pH.

**Table 1:** Patient characteristics

	DKA (*n* = 21)	Non-DKA (*n* = 50)	*P* value
Age (years) median (IQR)	44 (29–48)	53 (41–62)	0.02
Males *n* (%)	12 (57)	27 (54)	0.03
IDDM *n* (%)	12 (57)	12 (24)	0.02
CKD *n* (%)	2 (9)	12 (24)	0.13

*DKA* diabetic ketoacidosis, *CKD* chronic kidney disease (baseline glomerular filtration rate less than 60 ml/min), *IQR* interquartile range

**Table 2:** Outcome variables

	DKA (*n* =21)	Non-DKA (*n* = 50)	*P* value
Glucose (mg/dL)	804 (650–897)	592 (564–675)	0.003
ETCO2 (mm Hg)	17 (9–27)	37 (34–41)	<0.001
pH	7.16 (7.01–7.27)	7.38 (7.33–7.43)	<0.001
pCO2 (mm Hg)	25 (15.0–29.7)	41.75 (37.0–47.0)	<0.001
Bicarbonate (mmol/L)	9 (5–15)	24 (22–25)	<0.001
Beta-Hydroxybutyrate (mmol/L)	11.35 (7.9–14.0)	0.37 (0.17–1.21)	<0.001

Data presented as median (IQR). *ETCO2* end tidal carbon dioxide, *pCO2* partial pressure of carbon dioxide, *CKD* chronic kidney disease with baseline glomerular filtration rate less than 60 ml/min, *IDDM* insulin dependent diabetes mellitus

### Main results

**Fig. 1:**
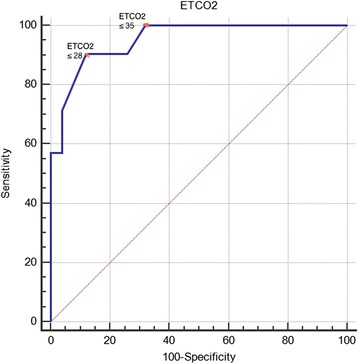
ROC curve showing overall diagnostic accuracy of ETCO2

The diagnostic performance of ETCO_2_ in diagnosing DKA is represented in the ROC in Fig. 1. The area under the curve [AUC] was 0.95 (95 % CI, 0.91 to 0.99). Test sensitivity at ETCO_2_ level ≥35 mmHg was 100 % (95 % CI, 83.9–100) and specificity was 68 (95 % CI, 53–80). At an ETCO_2_ level less than or equal to 21 mmHg specificity was 100 % (95 % CI, 92.9–100.0) and sensitivity was 57 % (95 % CI, 34–78). Table 3 provides the sensitivities and specificities for DKA at different ETCO_2_ cut-offs.

**Table 3:** Sensitivities and specificities of end tidal capnography

Criterion	Sensitivity	95 % CI	Specificity	95 % CI	+LR	95 % CI	-LR	95 % CI
≤21	57.14	34–78.2	100.00	92.9–100.0	0.00		0.43	0.3–0.7
≤23	57.14	34–78.2	98.00	89.4–99.9	28.57	4.0–206.0	0.44	0.3–0.7
≤25	57.14	34–78.2	96.00	86.3–99.5	14.29	3.5–58.4	0.45	0.3–0.7
≤26	71.43	47.8–88.7	96.00	86.3–99.5	17.86	4.5–71.3	0.30	0.2–0.8
≤27	85.71	63.7–97.0	90.00	78.2–96.7	8.57	3.7–20.0	0.16	0.06–0.5
≤28	90.48	69.9–98.8	88.00	75.7–95.5	7.54	3.5–16.2	0.11	0.03–0.4
≤31	90.48	69.9–98.8	86.00	73.3–94.2	6.46	3.2–13.0	0.11	0.03–0.4
≤32	90.48	69.9–98.8	84.00	70.9–92.8	5.65	3.0–10.8	0.11	0.03–0.4
≤33	90.48	69.9–98.8	80.00	66.3–90.0	4.52	2.6–8.00	0.12	0.03–0.4
≤34	90.48	69.9–98.8	74.00	59.7–85.4	3.48	2.1–5.7	0.13	0.03–0.5
≤35	100.00	83.9–100	68.00	53.3–80.5	56.8	2.1–4.7	0.00	

**Fig. 2:**
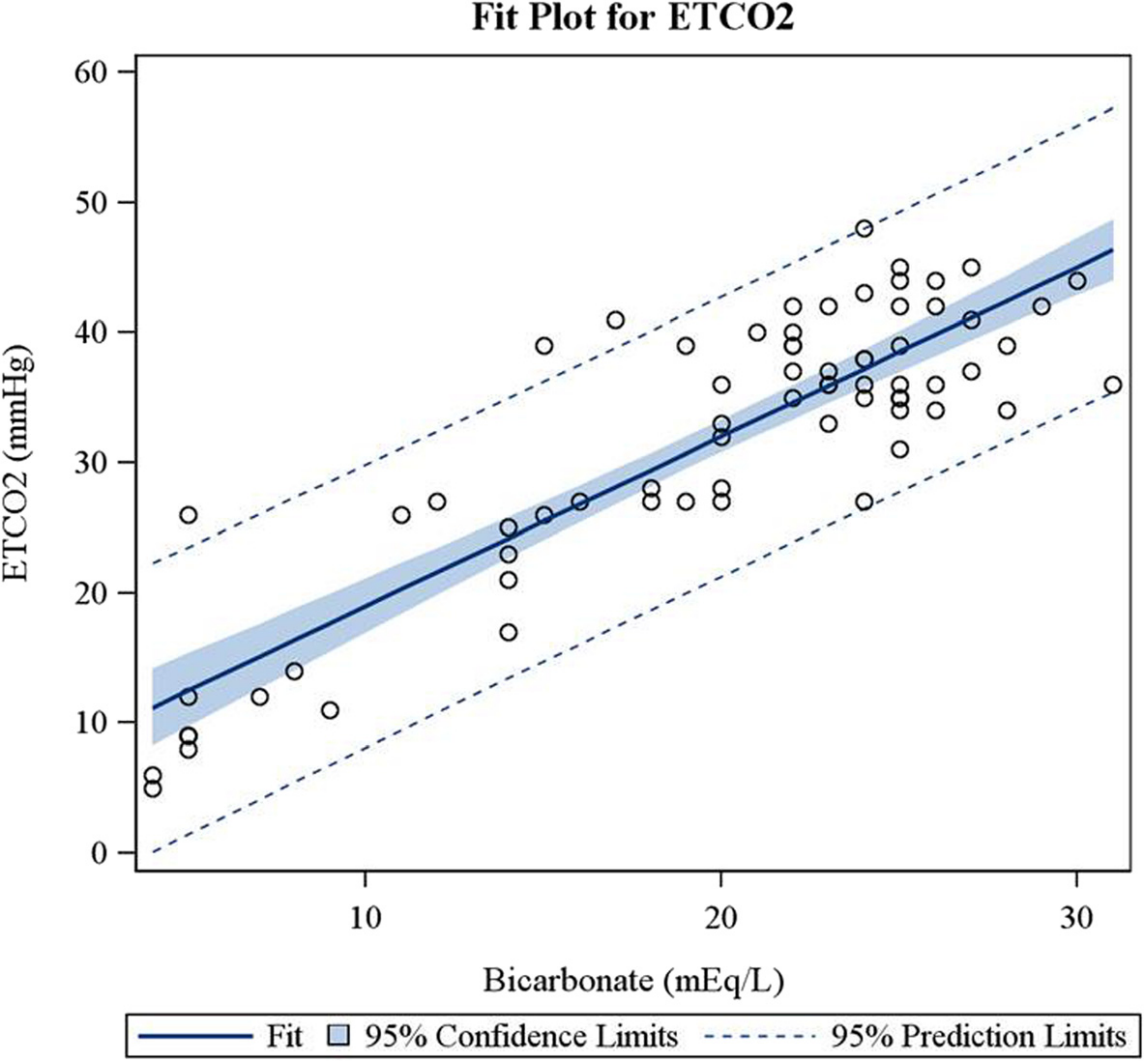
ETCO2 and bicarbonate correlation fit plot

**Fig. 3:**
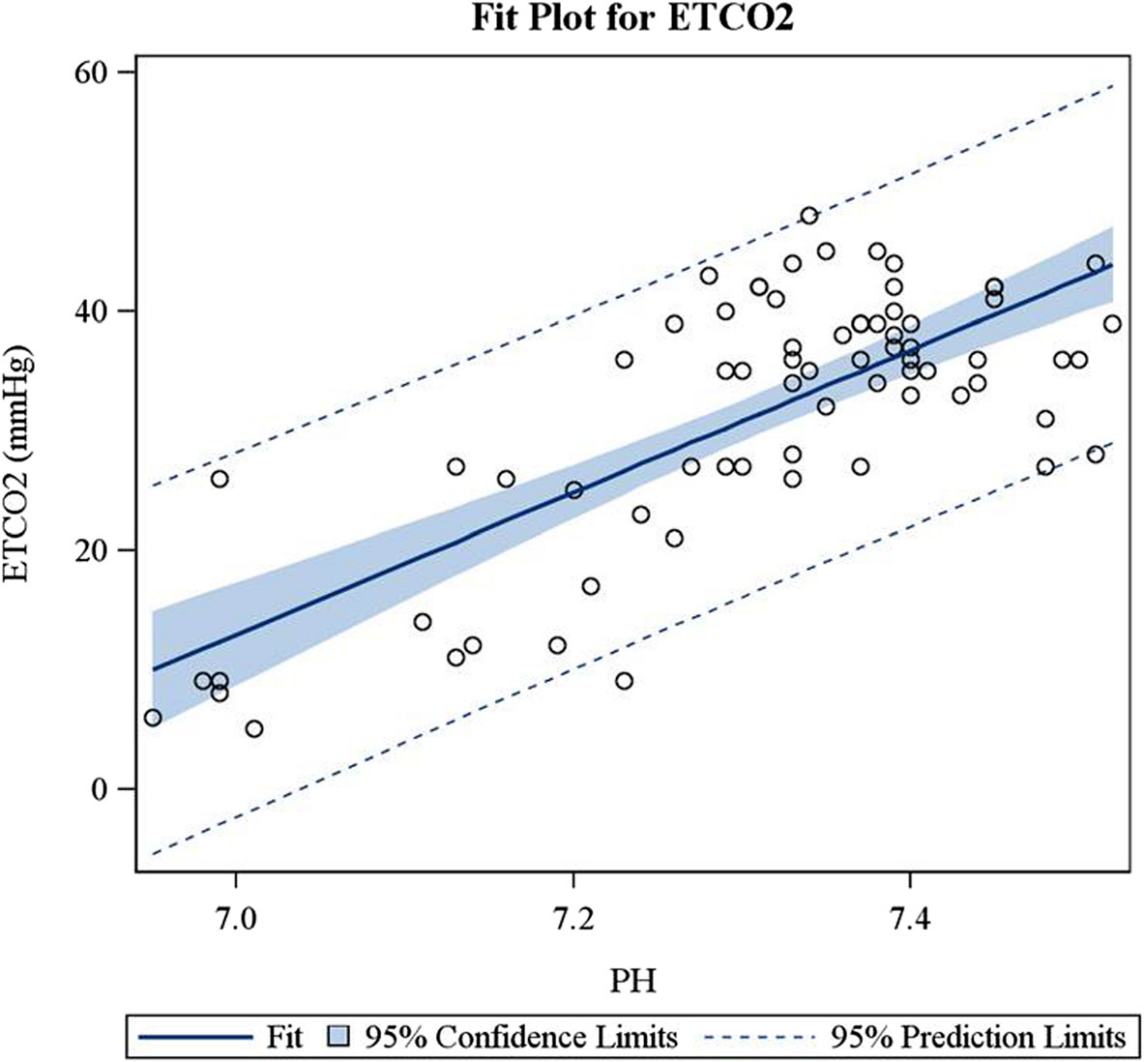
ETCO2 and venous blood gas PH correlation fit plot

Finally, there was a strong correlation between ETCO_2_ and serum bicarbonate levels (*r* = 0.88, *P* < .0001), coefficient of determination r^2^ = 0.76 (Fig. 2); and between ETCO_2_ and VBG pH (*r* = 0.75, *P* < .0001), coefficient of determination r^2^ = 0.56 (Fig. 3). There was also a strong correlation between the VBG pH and measured serum bicarbonate (*r* = 0.84, *P* < 0.0001), coefficient of determination r^2^ = 0.70.

## Discussion

DKA is one of the few endocrine emergencies. It is characterized by hyperglycaemia, ketonemia and metabolic acidosis along with electrolyte abnormalities. It is a condition that should be readily recognized and promptly treated. The mainstay of medical treatment is intravenous fluid rehydration, insulin replenishment through an intravenous drip, electrolyte correction and identification of the cause of the ketotic state [1].

DKA and severe hyperglycaemia are distinct clinical entities with different treatments. Although the mainstay treatment is fluid resuscitation, patients with DKA require insulin to reverse their ketotic state. Patients with severe hyperglycaemia without ketosis require fluid resuscitation first and foremost; they may or may not need insulin, and those who do infrequently require continuous insulin infusion [6]. We sought to study the test characteristics of ETCO_2_ in those presenting with blood sugar greater than 550 mg/dL in an attempt to identify thresholds which may help identify those with or without concomitant DKA.

In this cohort of adult patients presenting with high glucose, ETCO_2_ exhibited a good diagnostic performance (AUC 0.95), 95 % CI, 0.91 to 0.99. DKA was highly prevalent in patients with glucose > 550 mg/dL (prevalence 30 %). At ETCO_2_ ≤ 21 mmHg, DKA was ruled in (specificity 100 %) At ETCO_2_ ≥ 35 mmhg, DKA was ruled out (sensitivity of 100 %). Furthermore, there was a strong correlation between ETCO_2_ and PH as well as bicarbonate, which would suggest that ETCO_2_ could be useful in identifying DKA patients presenting with remarkably low PH levels and would allow faster and more aggressive care.

Our study differs from other studies in several ways. First, our cohort consisted of patients with blood glucose > 550 mg/dl measured by glucometer. Second, we used VBG pH to diagnose acidemia. Several studies have shown that venous pH and pCO_2_ correlated highly with arterial pH and pCO_2_ [7–9]. In patients with DKA, Ma et al. demonstrated that venous and arterial pH have also high agreement (mean bias −0.015 ± 0.006 pH units) [10]. In this study, we also found a strong correlation between the VBG pH and measured serum bicarbonate (*R* = 0.8395, *P* < 0.0001). Therefore, it is reasonable to replace the arterial blood gas with venous blood gas in the evaluation of a patient with DKA. Third, we used ßHB level >3 mmol/L to diagnose ketonemia. Although the American Diabetes Association (ADA) [11] states that urinary ketones are not reliable and that measurement of ßHB is preferred, no specific diagnostic cut-offs are mentioned. Studies that examined the correlation between serum bicarbonate and ßHB in DKA patients determined that a serum ßHB level <3 mmol/L does not correlate with an acidotic state [12, 13]. Fourth, our study was conducted in adult patients. Most of the studies that have evaluated the relationship between ETCO_2_ and acidosis were in pediatric patients with type I diabetes [14–16]. These studies have shown a very high diagnostic accuracy in pediatric patients. Both Fearon et al. [14] and Gilhotra et al. [16] showed ETCO_2_ values less than 29 mmHg could identify DKA with a specificity of 100 and 91 %, respectively, whereas no patient with ETCO_2_ values > 35 mmHg had DKA (sensitivity 100 %). In contradistinction, in this study, the corresponding specificity at ETCO_2_ values < 29 mmHg was only 88 % (95 % CI, 75–95), however, the sensitivity at ETCO_2_ values > 35 mmHg was also 100 % (95 % CI, 84–100). The different diagnostic accuracies at the same ETCO_2_ cut-offs between our study and the paediatric studies may be secondary to different spectrum and distribution of acute or chronic acid base disorders in adults compared to the paediatric population or secondary to statistical variation.

To our knowledge, only one study by Soleimanpour et al. has evaluated the utility of ETCO_2_ in adults as a screening tool for DKA with blood glucose > 250 mg/dL and clinical symptoms of DKA [17]. The study was conducted in an emergency department in Iran. The mean glucose levels were 458 mg/dL in DKA patients and 362 mg/dL in non-DKA patients. The Soleimanpour study showed that ETCO_2_ had a uniquely high diagnostic accuracy (AUC 0.963) overall, and at ETCO_2_ cut-off of 24.5 mmHg; it was highly sensitive (90 %) and specific (90 %). The diagnostic performance of our test as measured by AUC was very high (AUC 0.95). Specificity at ETCO_2_ cut-off of 24.5 in our study was slightly higher (96 % vs. 90 %); however our study did not demonstrate a high sensitivity at this ETCO_2_ cut-off (sensitivity 57 % vs. 90 %). Several patients in our cohort had DKA at higher ETCO_2_ levels than 24.5 either because of chronic respiratory acidosis disorders or because of coexisting metabolic alkalosis.

### Limitations

Our study has some limitations. First, the results can only be applied to patients with blood glucose >550 mg/dL (30.6 mmol/L). It is important to note that patients with blood glucose level <550 mg/dL can be in DKA, however we chose not to look at this subset of population since it was studied earlier in the literature and because our main objective was to check the test characteristics of the test among patients with severe hyperglycemia [17]. A non-invasive test with a strong positive predictive value for DKA would also be useful to identify and urgently treat those with glucose levels that might not otherwise be concerning. Second, although the study was adequately powered to show that the AUC was >0.50, it remains a relatively small study with wide CIs around the sensitivity and specificity of any of the cut-off points. A more precise estimate of these cut-off points can be obtained by a larger sample size. Third, it would have been helpful to see the AUC for respiratory rate and compare it to the AUC for ETCO_2_ and perform a cost analysis for the use of ETCO_2,_ however, this was not the aim of this study and it is our hope that this study can stimulate future research studies. Finally, It would also have been important to provide the data on other potential confounders of ETCO_2_ within our patient population, however a significant amount of our patients did not have any formal diagnosis of Chronic Obstructive Pulmonary Disease (COPD) or any Pulmonary Function Tests (PFTs) in our records.

## Conclusion

This single centre study suggests that end tidal capnography in adults presenting to the emergency department with glucose levels > 550 mg/dL may useful in identifying and ruling out DKA. The external validity of the results should be confirmed by further prospective studies.

## Ethics

This study was approved by Henry Ford Hospital’s institutional review board (IRB #8560). All procedures performed in studies involving human participants were in accordance with the ethical standards of the institutional review board at our institution and with the 1964 Helsinki declaration and its later amendments or comparable ethical standards.

## Consent

A waiver of informed consent was granted by our IRB. The authors have obtained consent to publish data from participants.
